# Are Drug Companies Living Up to Their Human Rights Responsibilities? Moving Toward Assessment

**DOI:** 10.1371/journal.pmed.1000310

**Published:** 2010-09-28

**Authors:** Sofia Gruskin, Zyde Raad

**Affiliations:** Program on International Health and Human Rights, Department of Global Health and Population, Harvard School of Public Health, Boston, Massachusetts, United States of America

## Abstract

The human rights responsibilities of drug companies have been considered for years by nongovernmental organizations, but were most sharply defined in a report by the UN Special Rapporteur on the right to health, submitted to the United Nations General Assembly in August 2008. The “Human Rights Guidelines for Pharmaceutical Companies in relation to Access to Medicines” include responsibilities for transparency, management, monitoring and accountability, pricing, and ethical marketing, and against lobbying for more protection in intellectual property laws, applying for patents for trivial modifications of existing medicines, inappropriate drug promotion, and excessive pricing. Two years after the release of the Guidelines, the *PLoS Medicine* Debate asks whether drug companies are living up to their human rights responsibilities. Sofia Gruskin and Zyde Raad from the Harvard School of Public Health say more assessment is needed of such responsibilities; Geralyn Ritter, Vice President of Global Public Policy and Corporate Responsibility at Merck & Co. argues that multiple stakeholders could do more to help States deliver the right to health; and Paul Hunt and Rajat Khosla introduce Mr. Hunt's work as the UN Special Rapporteur on the right to the highest attainable standard of health, regarding the human rights responsibilities of pharmaceutical companies and access to medicines.

## Abstract 2

As one viewpoint of three in the *PLoS Medicine* Debate on whether drug companies are living up to their human rights responsibilities, Sofia Gruskin and Zyde Raad argue that companies' actions to promote access to medicines, including their interactions with state and non-state actors, must be better monitored.

*This is the first of three viewpoints examining the question of whether pharmaceutical companies are living up to their human rights responsibilities.*

## The Right to Health and Essential Medicines

Debate on the extent to which drug companies have human rights obligations has elicited differing and sometimes contradictory positions. As shorthand, two extremes can be cited. On one hand, some multinational drug companies claim to support human rights because of their philanthropic efforts such as large-scale drug donation programs or the adoption of corporate social responsibility (CSR) frameworks that promote, at least rhetorically, fair labor practices and nondiscrimination in the workplace [1],[2]. On the other hand, some activist groups argue that drug companies are by their very nature constantly violating health-related human rights, prioritizing patents and profits over people's access to essential and other medicines [3].

Both examples presuppose a common definition and understanding of human rights, and as advocacy positions, these need not be grounded in law *per se*. Yet efforts to credibly assess drug companies' compliance with human rights norms ought to be rooted in international legal agreements, or at least in their commonly agreed upon interpretations. What international law actually says is important. Perhaps most pertinent is the legally established human right to essential medicines (EM) as a derivative of the broader right to health [4]. Under the right to health, governments have the legal obligation to ensure that EM are available, accessible (including financially accessible or affordable), acceptable, and of appropriate quality, and are advised to develop Essential Medicines Lists using the WHO Model List as guidance. Moreover, they must prevent rights violations committed by non-state actors (NSAs)—including drug companies—in their jurisdictions to ensure these actions do not result in inappropriate restrictions of access to EM [4]. Examples cited include anti-competition legislation that promotes the use of generic drugs and the reduction of taxes imposed on generics [5]–[7].

What does this mean for drug companies or other NSAs like the Bill & Melinda Gates Foundation? Surprisingly, even as the accountability of governments under international human rights law for making EM available is increasingly well recognized [4], to date the relevant legal obligations of the pharmaceutical industry and other NSAs have been inadequately addressed. Even as concerns about access to medicines have begun to shed light on the human rights roles and responsibilities of the pharmaceutical industry, this discourse has been primarily in relation to intellectual property and trade law. Given the pharmaceutical industry's strong influence on access to medicines and health systems more generally [8], their specific human rights duties, as well as those of other NSAs, require more attention.

As an increasingly important framework for government accountability, the right to health is legally understood to encompass only the *responsibilities* of NSAs. These responsibilities are distinct from the legally binding *obligations* of states who sign on to human rights treaties. At first glance it might seem appealing to also assign legally binding obligations to drug companies, for example by encouraging them to sign on to human rights treaties, but it is not clear that encouraging them to assume obligations and powers on par with governments is a good thing. While research and debate in this area is clearly needed, even within the currently recognized right to health responsibilities of NSAs, some determination can already be made as to whether drug companies are living up to their duties to support states in their obligation to make quality EM available.

From a rights perspective, the question is how can the actions of drug companies be more comprehensively assessed? Early attempts to elaborate on the responsibilities of NSAs, culminating in the 2003 UN Norms for Transnational Corporations [9],[10], give limited guidance. More recent efforts by Ruggie and Hunt to clarify the human rights roles of businesses, and of the pharmaceutical industry in particular, pave the way for much needed work in this area [11]–[14]. We posit that concrete assessment of the human rights responsibilities of drug companies can be undertaken with attention to top-down, bottom-up, and horizontal approaches.

## Moving Toward Assessment of Drug Companies' Human Rights Responsibilities: Three Approaches

Starting from the *top down*, one can look to see whether drug companies are working with States in the jurisdictions where they operate, not only to prevent direct violations of human rights but to help States meet their legal obligations to ensure access to EM. These activities can include promulgating and adhering to regulations concerning drug safety and quality, which take human rights considerations into account as well as minimizing any potential negative health impacts of intellectual property protections [15]. *Bottom-up* approaches can include the governance of public–private partnerships (PPPs) involving drug companies and civil society organizations, and assessing their impact on human rights and access to EM [16],[17]. These bottom-up approaches can also include analyses of drug companies' responses to the human rights groups' monitoring of their actions to ensure they are in line with legal norms and standards.

Working *horizontally* would examine efforts to bring together different stakeholders as partners in shared spaces to address issues around access to EM. Such efforts include the engagement of drug companies in international schemes such as UNITAID and the UN Global Compact and its working groups on human rights, anti-corruption, and related issues, where participatory dialogue between civil society actors, governments, and the private sector take place [18],[19]. The effectiveness of such voluntary self-regulatory regimes for protecting or advancing human rights to date has rightly been questioned with questions raised about poor enforcement and self-promoting participation [20]–[22]. But the lessons learned from these initial efforts can serve as a starting point for assessment and for building accountability in future CSR efforts such as the benchmarking of drug companies' CSR activities with explicit attention to human rights [23]. Horizontal approaches to assessment would also be concerned with the extent of the engagement of drug companies and other NSAs in PPPs, and the extent to which these partnerships use human rights operationally to advance access to EM and to overcome obstacles limiting PPP success. For instance, attention to community participation and empowerment that is supported by human rights approaches may not only have short-term benefits for the partners involved, but also contribute in the long-term to local community ownership and sustainability after partnership resources have been exhausted [16],[17],[24],[25].

Lack of access to medicines continues to plague the health of millions living in resource-poor settings. Attention to the pharmaceutical industry's health-related human rights responsibilities has focused heavily on intellectual property, international trade law, and the ultimate costs of drugs—ignoring many dimensions of pharmaceuticals' wide range of influence on health systems and services, including national drug policies [26]–[28]. A better understanding of the pharmaceutical industry's responsibilities for advancing health-related human rights, and how these are shaped by interactions with states and NSAs, is critical to assessing whether drug companies are living up to their human rights responsibilities.

## Abbreviations

CSR: corporate social responsibility
EM: essential medicines
NSA: non-state actor
PPP: public–private partnership
